# Investigating remote assessments and interventions for multilingual people living with dementia and their caregivers

**DOI:** 10.1002/alz.086821

**Published:** 2025-01-09

**Authors:** Mohamed Taiebine, Louise Keegan

**Affiliations:** ^1^ Euro‐Mediterranean University of Fez (UEMF), Fez, Fez Morocco; ^2^ Moravian University, PA, PA USA

## Abstract

**Background:**

Given the widespread of tele‐assessment and tele‐rehabilitation in speech language pathology and clinical neuropsychology for monolingual English‐speaking patients with acquired neurogenic language and cognitive disorders, there is an urgent need to implement a culturally and linguistically tailored telepractice for multilingual people living with dementia (MPLWD), for whom there is no consensus on a standard model. This study aims to investigate the delivery model of remote assessment and intervention for this population.

**Method:**

A systematic scoping review was conducted in December 2023 following frameworks described by Arksey and O’Malley (2007). A literature search was conducted for articles published in English that addressed telepractice services MPLWD in the field of speech language pathology and clinical neuropsychology. Factors that contributed to the successful delivery of telepractice sessions were described in terms of technological medium, modality of intervention (assessment vs rehabilitation), as well as target population (patients, caregivers or both).

**Result:**

A total of 53 articles were identified through search engines and 12 duplicates were excluded. After title and abstract screening, another 32 studies were removed. Finally, a total of 9 full‐text studies were selected. This review has demonstrated a need for setting up tele‐rehabilitation for the benefit of MPLWDs. Despite the paucity of studies addressing multilingual considerations and needs of patients with dementia and their caregivers, the focus on caregivers was reported in 7 studies using different remote services (web‐based intervention, mobile, draw‐care), while 2 studies reflected on gender and health disparities stemming from Latino, Korean, American and Australian contexts. MPLWDs may experience a mild or severe loss of one or more of their acquired languages as a result of the neurodegenerative trajectory of their disease. They may also present cognitive, communicative, and socio‐professional impairments impeding their daily life functioning.

**Conclusion:**

The scarcity of studies reflects a need for future research of MPLWD. More inclusive model of telepractice is the leitmotiv for cross‐linguistic studies in ethnically diverse population with dementia. The underrepresentation of multilingual telepractice should be taken into consideration and technological advances should be made accessible to diverse MPLWDs in different cultures and languages through equity and digital inclusion.

